# Radiation-sparing managements for cervical cancer: a developing countries perspective

**DOI:** 10.1186/1477-7819-4-77

**Published:** 2006-11-13

**Authors:** Myrna Candelaria, Lucely Cetina, Alicia Garcia-Arias, Carlos Lopez-Graniel, Jaime de la Garza, Elizabeth Robles, Alfonso Duenas-Gonzalez

**Affiliations:** 1 Division of Clinical Research, Instituto Nacional de Cancerología. Mexico City, Mexico; 2 Department of Gynecology Oncology, Instituto Nacional de Cancerología. Mexico City, Mexico; 3 Unidad de Investigación Biomédica en Cáncer. Instituto de Investigaciones Biomédicas, Universidad Nacional Autónoma de México. Instituto Nacional de Cancerología. Mexico City, Mexico

## Abstract

Cervical cancer is the seventh most frequent cancer worldwide but more than 80% of cases occur in developing countries. Till date, radiation therapy with external beam and brachytherapy remains as the core treatment for most stages of cervical cancer. However, radiation treatment protocols and equipment modelled on the best developed countries can be seldom applied directly to developing countries owing to financial constraints and lack of qualified personnel, thus, a substantial proportion of patients do not have access to even palliative radiation therapy. Treatment options when the standard therapy is either not available or difficult to reproduce in particular settings is highly desirable with the potential to save lives that otherwise could be lost by the lack of adequate treatment. These options of treatment ideally had to have show, 1) that these are not inferior to the "standard" in terms of either survival or quality of life; 2) that these can be delivered in settings were the "standard" is not available or if available its quality is poor; and 3) that the treatment option be accepted by the population to be treated.

Based on these considerations, it is obvious that cervical cancer patients, particularly those who live in countries with limited resources and therefore may not have sufficient radiation therapy resources are in need of newer therapeutical options. There is now a considerable amount of information emanating from clinical studies where surgery has a major role in treating this disease. These forms of "radiation-sparing" treatments include total mesometrial resection that could make unnecessary the use of adjuvant radiation; neoadjuvant chemotherapy that could avoid the use of adjuvant radiation in around 85% of patients and preoperative chemoradiation that could make brachytherapy dispensable. The feasibility and therapeutical value of these potential forms of management need to be prospectively evaluated.

## Background

Cervical cancer ranks seventh in the list of most frequent cancers worldwide. However, this tumor is second only to breast cancer as the most common gynecological neoplasm. In addition, more than 80% of cases occur in developing countries [1] and in a typical developing country, 60% to 80% of invasive cases present locally advanced disease [2]. These figures are occurring despite cervical cancer is a preventable disease with a simple test such as pap smear. The issue of why pap smear-based screening campaigns have failed to reduce incidence and mortality in these countries is beyond the scope of the present review; however, it is vital to understand why countries with limited resources have been in general unable to successfully implement well-organized and high coverage campaigns. It seems then that screening methods may not equally work in all settings and underscore the need on performing research to find out which screening method would be the most appropriate for the specific social, educational, economical, political and geographic chararacteristics of countries [3].

In terms of treatment, invasive disease can be divided into three main groups: 1) early stage, which ranges from microinvasive disease IA1, IA2 to macroscopic disease confined to cervix and measuring <4 cm, IB1; 2) locally advanced FIGO stages IB2-IVA, and 3) IVB and recurrent disease.

## Treatment of early stage cervical cancer

The recommended treatment for IA1 patients is either a local procedure such as conization or total hysterectomy, depending on the patient's desire to remain fertile, whereas for IA2 patients the recommendation is for a radical hysterectomy which removes parametrial tissue, upper vagina and pelvic lymph nodes. On average, 8% of cases show positive pelvic lymph nodes. Because many women at this disease stage wish and deserve to preserve fertility, radical trachelectomy is becoming an option for these patients as well as for IB1 patients [4]. In surgically treated early-stage cases, the presence in the surgical specimen of a combination of intermediate-risk factors (vascular and lymphatic permeation, tumor size >2 cm, and deep cervical stroma invasion) or high-risk factors (positive pelvic lymph nodes, parametrial infiltration, and positive surgical margins) dictates the use of adjuvant radiation [5] or chemoradiation, respectively [7].

## Current concepts and treatment of locally advanced stages

Treatment of locally advanced cervical cancer experienced no major changes for the nearly 80 years during which exclusive radiation was considered the standard of care. However, over the last 20 years, a number of trials testing concurrent chemoradiation were performed in an attempt to improve treatment results. Despite this, in 1996 a National Institutes of Health Consensus Statement on cervical cancer stated that there was no evidence that hydroxyurea or any other concomitant chemotherapeutic agent should be added to pelvic irradiation and incorporated into standard practice [8]. It was not until 1999 that five randomized studies including nearly 2,000 patients were published, demonstrating that survival rate with concomitant chemotherapy (RT/CT) based on cisplatin was superior than that obtained with radiation alone [2].

Afterwards, a meta-analysis based on 19 trials (17 published and two unpublished) including 4,580 patients corroborated these findings, confirming that chemoradiation offers an absolute survival benefit of 12% at 5 years [9]. Thus, cisplatin-based chemoradiation was largely accepted as the standard of care for patients with cervical cancer whose treatment required radiation, except for patients with co-morbidities who are radiated for stage IB1 or less. An update of the aforementioned meta-analysis that includes 24 trials (21 published, three unpublished) and 4,921 patients strongly suggests that chemoradiation improves overall survival and progression-free survival, whether or not platinum was used, with absolute benefits of 10 and 13%, respectively. There was, however, statistical heterogeneity for these outcomes. There was some evidence that the effect was greater in trials including a high proportion of stage I and II patients. Chemoradiation also showed significant benefit for local recurrence and the suggestion of a benefit for distant recurrence. Acute hematological and gastrointestinal toxicity were significantly higher in the concomitant chemoradiation group. Treatment-related deaths were rare, but late effects of treatment were not well-reported; thus, the impact of chemoradiation on these effects could not be determined adequately [10].

Thus, the concurrent use of chemoradiation mostly based on cisplatin or cisplatin 5-fluorouracil has largely been accepted as the standard. In addition, this concurrent treatment has been shown to be reproducible in non-research setting. Several reported experiences of cisplatin chemoradiation as a routine management consistently show that the treatment in general is well tolerated and effective [11,12]. Current research efforts are by now mostly focused on how to increase the response rate therefore the use of radiosensitizer agents such as gemcitabine, taxanes, camptothecins and others either alone or in combination with cisplatin are undergoing testing in different phases. In this regard, gemcitabine is the newer cytotoxic agent with the most extensive evaluation. A randomized phase II trial demonstrated the superiority of the combination of standard cisplatin plus gemcitabine over cisplatin alone in terms of pathologic complete response rate, and an ongoing phase III trial that has completed accrual will eventually confirm these results regarding survival. On the other hand, several molecular targeted agents possessing radiosensitizing properties open the way for their testing either alone or with known cytotoxic radiosensitizers for cervical cancer. Among the latter, cetuximab, a monoclonal antibody against the epidermal growth factor receptor, and a combination of epigenetic therapy agents are being tested in patients with cervical cancer as an adjunct to chemoradiation with cisplatin [13].

## The situation of radiation resources in the developing countries

Radiotherapy is a multidisciplinary speciality that uses complex equipment and radiation sources for delivery of treatment. It is estimated that over 2100 megavoltage teletherapy machines are currently installed in developing countries. This figure is significantly below the estimated current needs of almost 5000 machines. A conservative estimate points to a need for about 10,000 machines by the year 2015. To worsen the picture not only occur limitations in equipments but also there is an enormous need for qualified professionals (including radiation oncologists, medical physicists, radiotherapy technicians, radiation protection officers, and maintenance engineers) capable of operating new radiotherapy equipment. Just as examples, only 22 out of 56 countries in Africa were known to have megavoltage therapy and the population served by each megavoltage machine ranges from 0.6 million to 70 million per machine. [14]. In the Asian Pacific countries as a whole, the situation was not much better, with significant deficiencies in the availability of all components of radiation therapy [15] as it is in Latin America where not only there is a shortfall of equipment, but also a major restriction to patient service is an insufficient number of specialists [16].

Cervical cancer in disadvantaged women accounts for 17% of all female cancers whereas in well developed countries, cervical cancer accounts for only 4% of female cancers [1]. These different patterns of cancer have a profound influence on the need for specific radiotherapy resources. These differences, coupled with the more advanced stages of cancer present in developing countries, place different demands on the selection and use of equipment for radiotherapy. The treatment protocols and equipment modelled on the best developed countries seldom can be applied directly to developing countries owing to financial constraints and lack of qualified personnel.

The treatment of cancer is an ever changing field always in the search for most effective therapies able to increase cure rate or at least to prolong survival as compared to the therapy considered at the time as standard. Nevertheless, survival can not always be the unique goal of therapies. Treatments that while have the same effect upon survival turn out to be less toxic or have better impact in quality of life are valuable. Lastly but not the least, to have treatment options when the standard therapy is either not available of difficult to reproduce in particular settings is highly desirable with the potential to save lives that otherwise could be lost by the lack of adequate treatment. These options of treatment ideally had to have shown, 1) that are not inferior to the "standard" in terms of either survival or quality of life; 2) that can be delivered in setting were the "standard" is not available of if available its quality is poor; and 3) that the treatment option be accepted by the population to be treated.

Based on these considerations, it is obvious that locally advanced cervical cancer patients, particularly those who live in countries with limited resources and therefore may not have sufficient radiation therapy resources are in need of newer therapeutical options. There is now a considerable amount of information emanating from clinical studies where surgery has the major role in treating this disease. These forms of "radiation-sparing" treatments are here discussed.

## Surgery alone without adjuvant radiation

There exists some lack of consensus on what early stage and locally advanced stages are. It seems that treatment preferences varies from Institution to Institution. Thus, in Institutions that routinely employ radical hysterectomy to treat IB1, IB2 and IIA stages the definition of locally advanced stages encompases from IIB to IVA as these later are treated with radiation or chemoradiation as definitive therapy. On the contrary, Institutions that use radiation or chemoradiation for IB2 and IIA cases defines locally advanced disease as stages IB2-IVA. In this context it is now clear that regardless of the Institutions treatment preference, the treatment early stage patients can be done by radical hysterectomy or definitive radiation. This evidence is supported by early trials. One of the first was a non-randomized trial that started in 1945 in the University of Michigan using either radical hysterectomy or radiation for stage I cases. In this trial 5-year survival was 90% with any one of these treatments [17]. The first randomized trial was performed between 1956 and 1966 in 119 patients with stage I carcinoma of the cervix by radical hysterectomy alone (58 patients) or radiotherapy alone (61 patients). Eighty-one per cent of the patients treated by radical hysterectomy survived 5 years as compared with 74 per cent of those treated by radiotherapy. The 10 year survival rates were 75 and 65 per cent, respectively, however, these differences were not statistically significant. Complications of treatment were relatively few except for a greater incidence of urinary tract problems after surgical treatment; most of these were resolved with restoration of normal function. Interestingly, since then it was showed that when recurrence occurred in the surgical series, further treatment by radiotherapy offered a reasonable prospect of survival however, radiotherapy failures left relatively little opportunity for later treatment by surgery [18]. The most recent randomized trial was reported by an Italian group in 1997, in which 343 eligible patients were randomised: 172 to surgery and 171 to radical radiotherapy. Adjuvant radiotherapy was delivered after surgery for women with surgical stage pT2b or greater, less than 3 mm of safe cervical stroma, cutthrough, or positive nodes. After a median follow-up of 87 (range 57–120) months, 5-year overall and disease-free survival were identical in the surgery and radiotherapy groups (83% and 74%, respectively, for both groups). Eighty-six women developed recurrent disease: 42 (25%) in the surgery group and 44 (26%) in the radiotherapy group [19].

Currently, the most accepted criteria for indicating adjuvant radiation alone is a combination of pathological findings that place the risk of recurrence as "intermediate". These are, according to Sedlis [5] the presence of 1) +CLS (capillary lymphatic space tumor involvement) plus SI (stromal invasion) of deep 1/3 with any tumor size; 2) +CLS plus SI of middle 1/3 and ≥2 cm of tumor size; 3) +CLS plus superficial 1/3 of SI and a tumor size ≥5 cm; and 4) -CLS plus deep of middle 1/3 with a tumor size ≥4 cm. In an update of the trial, investigators reported that radiation showed a statistically significant (46%) reduction in risk of recurrence and a statistically significant reduction in risk of progression or death (HR = 0.58, 90% CI = 0.40 to 0.85, p = 0.009) although the improvement in overall survival (p = 0.074) with radiation did not reach statistical significance [20]. On the other hand, it has also been demonstrated that for those cases who have in the surgical specimens factors confering high-risk of recurrence (positive pelvic lymph nodes, disease in parametria and positive surgical margins) adjuvant cisplatin-based chemoradiation improves progression-free and overall survival [7].

Less commonly, some institutions mainly from European countries and Japan also treat IIB cases with a primary radical hysterectomy. Although the results are in general terms roughly similar to those obtained with concurrent definitive chemoradiation with 5-year survival rates varying from 55% to 77%, the need for adjuvant radiation can be as high up to 72% of cases [21,22].

Within the context of this review regarding the issue of sparing radiation, it can be said that IB1 to IIA cases elected to be treated with a radical hysterectomy a substancial fraction of patients will require radiation as complementary treatment. The need of adjuvant radiation results from the major shortcoming of radical hysterectomy that is the failure within the treatment field. Höckel, analyzed the loco-regional relapse pattern of patients with high resolution magnetic resonance imaging and surgical exploration [23]. He found that most of the pelvic relapses arose at the dissection sites of the radical hysterectomy and appeared to originate from microscopic or occult tumor foci within the endopelvic surgical scar.

In rectal cancer, a significant improvement with respect to postoperative sequelae, loco-regional recurrences, and survival was achieved by the introduction of the total mesorectal excision, a high resolution sharp dissection of the rectum and its integrated mesentery based on developmentally defined topographic anatomy [24,25]. Based on this Höekel developed the total mesometrial resection (TMMR) for the treatment of cervical carcinoma [26-28] to reduce probability of local relapse and also to avoid the need of postoperative adjuvant radiation. Total mesometrial resection is a high resolution radical hysterectomy with autonomic nerve preservation based on developmentally defined surgical anatomy. TMMR is characterized by *en bloc *resection of the uterus, proximal vagina and mesometrium, the integral mesentery covered by intact bordering lamellae [29], transection of the supporting dense subperitoneal connective tissue [30] directly above the level of the exposed inferior hypogastric plexus, and extended pelvic/periaortic lymph node dissection preserving the superior hypogastric plexus. TMMR differs from the classical radical hysterectomy by consequent sharp instead of blunt separation of the parietal and visceral endopelvic planes; exposition of the complete mesometrium by separation from the bladder mesentery; separate dissection of the mesometrium and the dense subperitoneal connective tissue supporting the uterus and proximal vagina with minimal trauma instead of their composite division as parametrectomy with traumatic clamps (eg, Wertheim clamps); and exposition and mobilization of the superior hypogastric plexus, hypogastric nerves and proximal inferior hypogastric plexus.

In 1999, Höckel reported his results of a prospective trial in patients treated with TMMR for cervical carcinoma FIGO stages IB, IIA, and selected IIB. By July 2002, 71 patients with cervical cancer stages pT1b1 (48), pT1b2 (8), pT2a (3), pT2b (12) had undergone TMMR without adjuvant radiation. Fifty-four percent of the patients exhibited histopathologic high-risk factors. At a median observation period of 30 months (9–57 months) two patients relapsed locally, two patients developed pelvic and distant recurrences and two patients only distant recurrences. Three patients died from their disease. Grade 1 and 2 complications occurred in 20 patients, no patient had grade 3 or 4 complications. No severe long-term impairment of pelvic visceral functions related to autonomic nerve damage was detected. Based on these preliminary results, the author came to the conclusion that TMMR achieves a promising therapeutic index by providing a high probability of locoregional control at minimal short and long-term morbidity [31].

Taken together, TMMR as a variant of a classical radical hysterectomy has the potential to reduce the use of adjuvant radiation treatment and therefore could be suited for patients living in areas whose radiation resources are poor however, its value must be tested in randomized trials before adjvuant radiation can be safely avoided.

## Neoadjuvant chemotherapy followed by surgery without adjuvant radiation

The negative results of neoadjuvant chemotherapy followed by radiation [32], led to the general believe that induction or neoadjuvant chemotherapy is of no value in cervical carcinoma. However, a distinction must be made between the trials that use radiation or surgery to consolidate the response to neoadjuvant chemotherapy because in this tumor type, the modality of local treatment after induction chemotherapy matters. Despite the lack of randomized trials comparing the current standard chemoradiation versus radical surgery after induction chemotherapy, emerging data from a large number of neoadjuvant trials suggests that surgical resection after induction chemotherapy could be better as it bypass the cross-resistance between chemotherapy and radiation, thus, the disease remaining after chemotherapy, theoretically could be more effectively treated with surgery. Accordingly, randomized phase III trials have shown superior results in terms of survival when locally advanced (FIGO stages IB2-IVA) are treated with neoadjuvant chemotherapy followed by surgery as compared to radiation alone [33-36]. These results have also been analyzed in a meta-analysis based on individual patient data from 5 randomized trials conducted worldwide that included 872 patients and 368 deaths. The overall results show a highly significant benefit of this modality as compared to radiation alone, with a 36% reduction in the risk of death, which is equivalent to an absolute improvement in survival of 15% (8%–21%) at five years, increasing the survival from 45% to 60%. Similar risk reductions are observed for progression-free survival, and local and systemic control. This benefit appears to be of the same magnitud to that achieved with the new standard of cisplatin-based chemoradiation [32].

The results of the meta-analysis on this modality indirectly suggest that it is as effective as the current standard of cisplatin-based chemoradiation. It should be pointed out however, that the five studies included in the meta-analysis [32] used a chemotherapy based on cisplatin plus "old" drugs such as vincristine and bleomycin, which are clearly not the most effective as compared to the newer ones. In a comprehensive review on neoadjuvant chemotherapy followed by surgery performed by Eddy [37] it is shown that the response rates achieved with these regimens vary between 47% to 88%. On the contrary the response rate achieved with newer drug regimens incorporating taxanes [38,39], irinotecan [40], vinorelbine [41], and, gemcitabine [42,43] to platinum compunds seems higher. These data suggest that it could be possible to obtain better results if a highly effective drug combination is used as induction chemotherapy. Up to date, a direct comparison in a randomized phase III study between this modality with standard chemoradiation is lacking. We have recently reported a comparison between two consecutive phase II studies in patients with cervical carcinoma staged from IB2 to IIIB. In the first of these two studies, neoadjuvant cisplatin gemcitabine followed by surgery with no adjuvant radiation was employed and, in the second, the standard weekly cisplatin concurrent with pelvic radiation was used. Despite being a non-randomized comparison, both study groups were well-balanced for the most relevant clinico-pathological characteristics: histology, stage, tumor size, parametrial infiltration, performance status and pretreatment hemoglobin levels. Interestingly, at a median follow-up of almost three years, there are no differences in terms of survival [44], however, both arms are superior to an historical control treated with radiation alone [45]. Currently, the EORTC is conducting a multicentric phase III study to compare cisplatin-based neoadjuvant chemotherapy followed by surgery with or without adjuvant radiation versus standard cisplatin chemoradiation in stages IB2 to IIB.

Since we are discussing the issue on the ability of neoadjuvant chemotherapy followed by surgery to avoid or to spare the use of radiation postoperatively, the issue of the proper selection of patients and the maximum effort surgery to offer radical hysterectomy to most patients could not be underestimated. Pooling the results from a literature review of several neoadjuvant chemotherapy surgery trials, around 30% of the cases have been treated with adjuvant pelvic radiation [37]. However, the benefit of adjuvant radiation after surgery in locally advanced cases is unknown. Nowadays, the benefit shown of chemoradiation instead of radiation alone as adjuvant treatment of early staged patients with high-risk factors for recurrence [7] makes logical the employment of concurrent chemoradiation instead of radiation alone as adjuvant treatment for the locally advanced cases submitted to induction chemotherapy and radical resection which is feasible in this patient population [46]. Ideally, no adjuvant radiation (chemoradiation) should be needed without compromising the control of the disease if the goal is the avoid or to keep at minimum the use of radiation. In the current ongoing randomized comparison between neoadjuvant chemotherapy followed by surgery (cisplatin-based) against standard chemoradiaton in IB2-IIB patients, the resectability rate has approaching 100% and around 15% have required adjuvant or postoperative chemoradiation.

## Neoadjuvant or preoperative chemoradiation without brachytherapy

The triple modality of neoadjuvant concurrent chemoradiation followed by surgery is being studied not only in cervix uteri carcinoma but in other tumor types [47-51]. The rational behind preoperative chemoradiation is the obtaining of a major or complete pathological response, being known that one of the stronger predictors of longer survival is pathological complete response for almost any tumor type. Another benefit of this approach is that it allows one to compare the efficacy of the chemoradiation. For instance, chemoradiation previous to surgery produces favorable local control rates, often in excess of 90% for patients with localized and locally advanced extremity sarcomas [47]. In a study of 88 patients with advanced rectal cancer, preoperative chemoradiation therapy followed by definitive surgical resection resulted in decreased recurrence and an overall survival of 86% at median follow-up of 33 months [48]. This modality has also been evaluated in esophageal cancer. Meluch *et al*., treated 49 operable esophageal cancer with preoperative chemoradiation followed by esophagectomy, of whom 46% achieved pathological complete response whereas 20% had only microscopic residual [49] whereas in a study of stage III non-small cell lung cancer, 79% of 42 patients could undergo resection, and the five-year survival rate for all patients and for those who underwent resection were 36.5% and 45.5% respectively, being these figures superior to reports in literature [50].

Several preoperative chemoradiation studies have been performed in cervical carcinoma with excellent results in terms of disease-free and overall survival. Of note, some of these trials have only used external radiation with or without concurrent chemotherapy whereas other have used both external beam and brachyterapy before surgery. In the contex of this review on the avoidability of using brachytherapy without compromising the results on survival, we emphasize on this latter issue.

A current paradigm in the treatment of cervical cancer with radiation therapy is that intracavitary brachytherapy is an essential component of radical treatment of cervical cancer. On the other hand, despite that for many years many institutions routinely used adjuvant extrafacial hysterectomy for bulky exophytic or "barrel" shaped tumors, this procedure as been gradually abandoned due to the fact that a randomized study showed no benefit on survival of adjuvant hysterectomy, despite the study suggested that patients with tumors measuring 4 to 6 cm, may have benefitted from extrafascial hysterectomy [51].

No major research efforts however, have been placed on the role of a radical hysterectomy after definitive radiation, mainly due to the fact of the undemonstrated utility of extrafacial hysterectomy as above mentioned, except for a study reported in 1993 in which uterine cancer patients deemed to have at high-risk for recurrence underwent radical hysterectomy after definitive radiation. Authors conclude that radical hysterectomy after radiation is morbid but may be effective in treating patients with 1) large cervical tumors, 2) cervical cancer that responds poorly to radiation, 3) small recurrent cervical tumors, 4) patients unable to undergo brachytherapy for cervical cancer, and 5) uterine sarcomas involving the cervix [52].

Data from a few phase II studies of preoperative concurrent chemoradiation with no brachytherapy demonstrate excellent local control rates and survival. In a pilot study of 40 patients staged IB2 to IVA, Jurado *et al*., reported the results of preoperative cisplatin 5-fluorouracil during external beam radiotherapy to 45Gy with no intracavitary treatment, however, half of patients received intraoperative radiation. Interestingly, the 9-year local control, disease-free survival and survival were 86%, 81% and 85% respectively. As expected, those with pathological complete response (67.5%) fared better [53]. In a study from Italy, 26 patients (24, IIB and 2, IIIA) were treated with chemoradiation based on cisplatin and 5-fluorouracil during external radiation up to 39.6 Gy before radical surgery. A complete pathological response was observed in 54.2%, and a median follow-up of 33 months, the two-year local control was 91.7% [54]. In the aim to better assess the role of brachytherapy when radiation is followed by hysterectomy, we analyzed the pathological complete response rates in trials that used either external beam radiation with or without concurrent chemotherapy in regard to the use or not of brachytherapy. As inferred from Table 1, it seems that in the context of stages IB to IIB, brachytherapy adds nothing in terms of the probability of achieving pathological complete response. Overall, in trials using external beam radiation (EBRT) at doses between 37.4 to 52 Gy, in common fractions of 1.8 or 2 Gy daily, plus brachytherapy the average complete pathological complete response rate observed is 50% (41%, 44%, 48%, 48%, 69%) [51,55-58], whereas in those using EBRT at similar doses plus concurrent chemotherapy with either weekly cisplatin or the combination of cisplatin and 5-fluorouracil plus brachytherapy, the corresponding average is 51.1% (38%, 45%, 49%, 52%, 60% and 63%) [57-62]. Interestingly, in four trials (one of them with to arms) using EBRT with chemotherapy but no brachytherapy, the pathological response rate is essentially the same, a mean of 51.6% (45%, 45.2%, 46.6%, 54.2 and 67%) [16,53,63,64]. These data are quite provocative and suggest that for these not so advanced stages, brachytherapy could be dispensable, however, it must be stressed that such comparison is based on highly heterogeneous trials and as such data is only hypothesis generating.

**Table 1:** Pathological complete response rates in trials of preoperative radiation or chemoradiation with and without brachytherapy.

**# of pat**	**[ref]**	**EBRT+BT**	**EBRT/CT+BT**	**EBRT/CT no BT**	**path CR (%)**
66	[55]	37.4-40Gy+20Gy			69
61	[56]	46Gy CDDP+27Gy			44
360	[57]	20Gy+40Gy			48
168	[58]	45Gy+30Gy			41
123	[51]	45Gy+30Gy			48
					Mean (50.0%)
20	[59]		45Gy CDDP/5FU+20Gy		60
43	[57]		45Gy CDDP+15Gy		63
35	[60]		45Gy CDDP/5FU+20Gy		45
175	[58]		45Gy CDDP+30Gy		52
112	[61]		45Gy CDDP/FU+15Gy		49
175	[62]		44Gy CDDD+16Gy		38
					Mean (51.1%)
30	[63]			45Gy CDDP/5FU	46.6
100	[64]			39Gy CDDP/5FU	45.2
24	[53]			39Gy CDDP/5FU	54.2
40	[70]			50Gy CDDP	45
40	[70]			50Gy CDDP/GEM	67
					Mean (51.6%)

Most patients were IB2-IIA, some included IIB. Trials whose definition of complete pathological response encompased microscopic residual disease were not accounted.

This hypothesis, needs to be tested in phase III trials randomizing patients to brachyterapy or to radical hysterectomy after EBRT concurrent with chemotherapy. These trials can be fundamented on the fact that as in many tumors pathological complete response is an excellent surrogate marker for survival [65-68]. Regarding cervical cancer we analyzed this issue in a retrospective study where four cohorts of patients with locally advanced cervical carcinoma (stages IB2-IIIB) included prospectively in phase II protocols of either neoadjuvant chemotherapy with 1) cisplatin-gemcitabine, 2) oxaliplatin-gemcitabine, 3) carboplatin-paclitaxel or 4) concurrent EBRT with cisplatin or cisplatin-gemcitabine followed by radical hysterectomy. In this analysis, one-hundred and fifty three (86%) of the 178 patients treated within these trials, underwent radical hysterectomy and were analyzed. Overall, the mean age was 44.7 and almost two-thirds were FIGO stage IIB. Pathological response rates were as follows: Complete in 60 cases (39.2%), Near-complete in 24 (15.6 %) and partial in 69 cases (45.1%). A higher proportion rate of pCR was observed in patients treated with chemoradiotherapy compared with patients receiving only chemotherapy (p = 0.0001). A total of 29 relapses (18.9%) were documented. The pathological response was the only factor influencing on relapse, since only 4/60 (6.6%) patients with pCR relapsed, compared with 25/93 (26.8%) patients with viable tumor, either pNear-CR or pPR (p = 0.001). Overall survival was 98.3% in patients with pCR versus 83% for patients with either pNear-CR or pPR (p = 0.009) [69]. In an update (unpublished) of the results in the preoperative chemoradiation trial, the actuarial 5-year survival for the 40 patients treated with EBRT with cisplatin gemcitabine, is 95% [70]. An additional advantage of performing radical hysterectomy with pelvic lymph node dissection is the evidence that after definitive radiation (EBRT and brachytherapy) around 17% of patients are left with positive pelvic lymph nodes that go untreated if not are removed by surgery [61,71].

Yet this triple modality seems promisory regarding local control and survival, surgical complications, specifically lymphocysts, fistula and hydronephrosis are more frequent to that reported in patients undergoing either upfront hysterectomy [72,73] or after neoadjuvant chemotherapy [74]. This higher surgical complication rate of this modality, therefore, should be weighed against the potential benefit in terms of local and systemic control. In addition to these observations, the longer time needed to complete the overall treatment raises the need for quality-of life evaluations in future studies investigating the role of preoperative chemoradiation in the treatment of cervical cancer.

These results led us to launch a randomized trial that is ongoing where patients in both arms receive EBRT at dose of 50 Gy with 2 Gy fraction and are randomized to either radical hysterectomy or brachytherapy. So far, 34 and 38 are at one year of follow-up after completing treatment and there have been four failures (2 persistances, 1 local and 1 local/systemic) in the brachytherapy arm and only 1 systemic failure in the surgical arm. The final results of this randomized trial would eventually indicate whether or not brachytherapy can be dispensable in the management of IB2 to IIB FIGO stage cervical carcinoma. A summary of current treatment and proposed "radiation-sparing managements for IB2-IIB patients is presented in Table 2.

**Table 2:** Summary of current treatment and proposed radiation-sparing managements for IB2-IIB patients.

**Procedure**	**Radiation need**
*Radical Hysterectomy +Adjuvant EBRT +/- brachy*	30–79%*
*Definitive chemoradiation*	100%*
Total Mesometrial Resection	0%
Neoadjuvant chemotherapy/Surgery	15%
Preoperative chemoradiation**	0% (brachytherapy)

* Current treatments in italics; ** All require EBRT

## Conclusion

As for now, external beam radiation and brachytherapy remain as the core treatment for most stages of cervical carcinoma. Of late, the results obtained with radiation were improved by adding concurrent chemoradiation which has been widely adopted as the newer standard of management around the world. However, in many developing countries cervical cancer patients may go untreated or receive a suboptimal therapy due to lack or poor radiation resources. In this situation, it makes sense to study the here called "radiation sparing managements" such as the total mesometrial resection thal could make unnecessary the use of adjuvant radiation; the use of neoadjuvant chemotherapy that could avoid the use of adjuvant radiation in around 85% of patients as well as preoperative chemoradiation that could make brachytherapy dispensable. Nevertheless, these alternatives for management of cervical cancer that decrease or avoid the use of radiation are far from solving the problem. Access not only to radiation but to cancer treatments is one of the areas of greatest need in the developing word, particularly for the countries with the lowest-revenue such as those in the sub-Saharan Africa. If neoadjuvant chemotherapy or preoperative chemoradiation is contemplated, in most low and middle-income countries the cost of drugs is usually covered by patients who may found it prohibitive. A similar situation can be encountered regarding the availability of local or regional cancer centers equiped with surgical rooms and intensive care units required for radical surgical procedures. In both cases, qualified medical and surgical oncologists may also be insufficient or unavailable at all.

Much has been written on cancer initiatives for the developing word. Clearly, the expanding burden of cancer is a problem that requires a concerted global responses. While cancer control in developing world is an ambitious long-term goal, the availability of treatment alternatives suited to specific settings may represent small steps toward that end.

## Competing interests

The author(s) declare that they have no competing interests.

## Authors' contributions

MC, LC, AG-A, CL-G, JG and ER contributed in compiling, analyzing and discussing information for the manuscript; AD-G wrote the manuscript. All authors participated in conceiving the manuscript and critically read it.
